# Risk of ischemic stroke in metabolically healthy obesity: A nationwide population-based study

**DOI:** 10.1371/journal.pone.0195210

**Published:** 2018-03-30

**Authors:** Hyun-Jung Lee, Eue-Keun Choi, Seung-Hwan Lee, Yong-Jin Kim, Kyung-Do Han, Seil Oh

**Affiliations:** 1 Department of Internal Medicine, Seoul National University Hospital, Seoul, Republic of Korea; 2 Division of Endocrinology and Metabolism, Department of Internal Medicine, Seoul St. Mary’s Hospital, College of Medicine, The Catholic University of Korea, Seoul, Republic of Korea; 3 Department of Biostatistics, College of Medicine, The Catholic University of Korea, Seoul, Republic of Korea; University College London, UNITED KINGDOM

## Abstract

**Background:**

Whether metabolically healthy obese (MHO) individuals are at increased risk of ischemic stroke is not well known. We investigated the association of the MHO phenotype with ischemic stroke.

**Methods:**

A total of 354,083 adults (age 45.8 ± 14.2 years) from the Korean National Health Insurance Service–National Sample Cohort enrolled in 2004–2008 were followed-up for incident ischemic stroke until 2013. Subjects meeting none of the metabolic syndrome criteria were classified as ‘metabolically healthy’. The cohort was categorized into four groups according to obesity and metabolic status: metabolically healthy normal weight (MHNW), metabolically unhealthy normal weight (MUNW), MHO, and metabolically unhealthy obese (MUO).

**Results:**

Ischemic stroke was newly diagnosed in 4,884 (1.4%) individuals during a mean follow-up of 7.4 ± 1.5 years. Stroke incidence rates for the MHNW, MUNW, MHO, and MUO groups were 0.56, 2.61, 0.61, and 2.76 per 1,000 person-years, respectively. While risk for stroke increased significantly in metabolically unhealthy groups, it was not increased in MHO compared to the MHNW group on multivariate analysis. In metabolically healthy individuals, obesity did not increase the risk for ischemic stroke, regardless of the severity of obesity. Meanwhile, in metabolically unhealthy individuals, being obese was significantly associated with increased risk of stroke.

**Conclusions:**

MHO individuals were not at increased risk for ischemic stroke. However, obesity increased risk for ischemic stroke in persons with metabolic risk factors; therefore, maintaining normal weight may be more important for this population. Also, metabolic unhealthiness showed greater association than obesity with stroke.

## Introduction

Obesity and metabolic syndrome, often co-existing, are associated with increased cardiovascular risk and poor health outcomes[1–4]. Obesity is excess adiposity, which correlates with excess body weight. Body mass index (BMI) is an easily obtained measure of obesity which represents excess body weight, and shows strong association with mortality and cardiovascular prognosis in previous studies[1, 4]. Other measures of obesity include fat mass (%), waist circumference, or waist-to-hip ratio, but BMI over 30 or 25 kg/m^2^ according to ethnicity is the most used and validated measure[5–8]. However, a subset of obese individuals with an otherwise metabolically healthy profile, termed the ‘metabolically healthy obese (MHO)’, may have a better cardiovascular prognosis compared to the rest of the obese population. Defining ‘metabolically healthy’ is a matter of debate, and depending on studies, having 0 or up to 1 metabolic syndrome risk factor is the commonly used definition[9].

Stroke is a major health burden despite decreasing mortality[10]. Stroke is often considered as part of a composite cardiovascular outcome with coronary heart disease. However, risk for stroke and risk for coronary heart disease may be different. Some previous studies have found MHO to be at increased cardiovascular risk (including stroke)[11–13], while others have found no difference in risk between the MHO and MHNW phenotypes[14–17]. Even when examining stroke alone as the primary outcome, there were conflicting data that stroke risk was increased[11] and not increased[14, 15] in MHO.

We examined the risk for ischemic stroke associated with obesity and metabolic health status, using a strict definition for being metabolic healthy, in a nationwide population-based cohort.

## Materials and methods

### Study population

The Korean National Health Insurance Service–National Sample Cohort (NHIS-NSC) consists of 1,025,340 randomly selected subjects from the general Korean population in 2002 (2.2% of the total Korean population) and their follow-up data until December 2013. Details of this database representing the general Korean population have been previously described[18–20]. Demographics, medical treatment records of inpatient and outpatient care such as diagnoses, prescriptions, and procedures, and nationwide health examination results were available for research.

From the NHIS-NSC, a retrospective cohort of Korean adults (age ≥ 20 years), who had undergone a baseline health examination including body mass index (BMI) from 2004 to 2008 and did not have a prior diagnosis of ischemic stroke, was extracted (n = 370,537). Those with a previous diagnosis of ischemic stroke (I63-64) during the past 3 years were excluded, because previous diagnostic coding of stroke in the NHIS claims database has limitations to differentiate from new-onset stroke: this excluded group was nearly 20 years older as well as being more obese, and having more underlying disease. Underweight individuals (BMI < 18.5 kg/m^2^) were also excluded (n = 16,454), as underweight individuals have shown increased risk for morbidity and mortality compared to normal weight individuals[6, 21, 22]. A total of 354,083 were included in the final study cohort.

Diagnoses were defined by the International Classification of Diseases, 10th revision (ICD-10) with record of hospitalization or outpatient clinic treatments. Definitions of covariates were validated in our previous studies[18, 23, 24], and are summarized in S1 Table. Demographic data were obtained by questionnaires at baseline health examinations. This study was approved by the Seoul National University Hospital Institutional Review Board, and adhered to the Declaration of Helsinki.

### Definitions of obesity and metabolic health

Obesity was ascertained by BMI. BMI was calculated by dividing weight in kilograms by the square of height in meters (kg/m^2^) and categorized using the WHO Western Pacific Region definition of obesity for Asians[25]: normal weight (BMI 18.5–24.9 kg/m^2^) and obese (BMI ≥ 25 kg/m^2^), with obesity being further classified as stage I (BMI 25–29.9 kg/m^2^) and stage II (BMI ≥ 30 kg/m^2^). Underweight individuals (BMI < 18.5 kg/m^2^) were excluded from the study.

Metabolic health status was ascertained using metabolic syndrome criteria[26]. As only total cholesterol levels were measured at baseline health examinations before 2009, neither triglycerides nor high-density lipoprotein-cholesterol (HDL) levels were available; thus, we used elevated total cholesterol levels of ≥ 240 mg/dL as a proxy indicator of abnormal lipid levels, which is the cut-off for high cholesterol levels according to NCEP-ATPIII and Korean guidelines[27, 28]. In concordance to a recently proposed harmonized definition of MHO[9], being metabolically healthy was defined as meeting none of the following metabolic syndrome criteria at baseline: i) elevated blood pressure (SBP ≥ 130 and/or DBP ≥ 85 mmHg) or treatment for hypertension, ii) elevated fasting glucose (≥ 100 mg/dL) or treatment for diabetes mellitus, iii) elevated total cholesterol (≥ 240 mg/dL) or treatment for dyslipidemia. Individuals who met one or more of the above criteria were considered metabolically unhealthy. The waist circumference criterion was excluded. Treatment for hypertension, type 2 diabetes mellitus, dyslipidemia were ascertained using diagnosis codes during the past year with record of hospitalization or outpatient visit (S1 Table).

The cohort was categorized into four groups according to obesity and metabolic status: metabolically healthy normal weight (MHNW), metabolically unhealthy normal weight (MUNW), MHO, and metabolically unhealthy obese (MUO).

### Endpoint

The primary endpoint was newly diagnosed ischemic stroke. This variable was defined as diagnosis of ischemic stroke (I63-64) given during hospitalization combined with claims for neurologic imaging by computed tomography or magnetic resonance[18, 29, 30]. Patients who did not develop ischemic stroke during the follow-up period were censored on the day of drop-out (due to death or emigration) or at the end of follow-up.

### Statistical analysis

The characteristics of the cohort are presented as means ± standard deviation (SD) for continuous variables and percentage for categorical variables. Differences between groups were tested by analysis of variance (ANOVA) for continuous variables and chi-square test for categorical variables.

Incidence rates were calculated per 1,000 person-years. The cumulative incidence of ischemic stroke for each group was plotted with Kaplan-Meier curves and compared by the log-rank test. Cox proportional hazard models were used to assess the risk of ischemic stroke. Time was defined as days from inclusion to either incident ischemic stroke or censoring due to death, emigration, or end of follow-up. Stroke risk was expressed as the hazard ratio (HR) with 95% confidence interval (95%CI). Multivariate adjustments were made for sex, age, income (lower 20 percentile, upper 80 percentile), area (urban, rural), smoking status (non-smoker, ex-smoker, current smoker), alcohol intake (none, 1–3 times/month, ≥ 1 time/week), exercise status (none, 1–4 times/week, ≥ 5 times/week), and comorbidities including components of CHA_2_DS_2_-VASc score such as ischemic heart disease, peripheral artery disease, congestive heart failure, transient ischemic attack, venous thromboembolism, as well as chronic obstructive pulmonary disease, end-stage renal disease, liver cirrhosis, cancer, and history of cardiac surgery. To examine the effect of BMI, we made further adjustments for metabolic components such as elevated blood pressure, elevated fasting glucose, and elevated cholesterol. To examine the effect of metabolic components, we made further adjustments for BMI. The significance level was set at two-sided p < 0.05. All statistical analyses were performed using SPSS version 22 and SAS version 9.2 (SAS Institute, Cary, NC, USA).

## Results

### Baseline characteristics of the study population

The baseline characteristics of the study population are summarized in Table 1. 31.9% of the participants were obese, though only 3.2% had BMI ≥ 30 kg/m^2^. More than half (61.2%) were metabolically unhealthy. Participants were categorized into four groups according to obesity and metabolic health status: MHNW (31.2%), MUNW (36.9%), MHO (7.5%), and MUO (24.4%). The groups showed significant differences in all baseline characteristics. Metabolically unhealthy groups tended to be older. The obese groups showed higher proportion of men. Metabolically unhealthy groups had higher rates of comorbidities compared to metabolically healthy groups. The MUO tended to have more metabolic risk factors compared to the MUNW. At the end of the follow-up period, a higher proportion of the MHO group compared to the MHNW group became metabolically unhealthy. In the MHNW group, 0.7%, 6.3%, and 11.6% developed diabetes, hypertension, and dyslipidemia, respectively; in total, 15.1% became metabolically unhealthy. On the other hand, in the MHO group, 2.4%, 13.0%, and 16.8% developed diabetes, hypertension, and dyslipidemia, respectively; in total, 23.5% became metabolically unhealthy.

**Table 1:** Baseline characteristics of the study population.

	Total(n = 354,083)	MHNW (n = 110,531, 31.2%)	MUNW (n = 130,583, 36.9%)	MHO (n = 26,448, 7.5%)	MUO (n = 86,521, 24.4%)
Male sex, n (%)	186,563 (52.7%)	46,599 (42.2%)	72,758 (55.7%)	15,395 (58.2%)	51,811 (59.9%)
Age, years	45.8 ± 14.2	39.1 ± 12.2	49.7 ± 14.5	41.7 ± 11.7	49.8 ± 13.4
Body mass index, kg/m^2^	23.8 ± 3.0	21.8 ± 1.7	22.5 ± 1.6	26.9 ± 1.8	27.4 ± 2.2
Elevated blood pressure*	146,622 (41.4%)		83,920 (64.3%)		62,702 (72.5%)
Elevated fasting glucose^†^	98,735 (27.9%)		58,419 (44.7%)		40,316 (46.6%)
Elevated total cholesterol^‡^	37,790 (10.7%)		20,514 (15.7%)		17,276 (20.0%)
Hypertension	52,487 (14.8%)		26,852 (20.6%)		25,635 (29.6%)
Diabetes mellitus	16,332 (4.6%)		8,590 (6.6%)		7,742 (8.9%)
Dyslipidemia	34,116 (9.6%)		18,613 (14.3%)		15,503 (17.9%)
Ischemic heart disease	10,246 (2.9%)	488 (0.4%)	5,069 (3.9%)	167 (0.6%)	4,522 (5.2%)
Peripheral artery disease	8,421 (2.4%)	555 (0.5%)	4,203 (3.2%)	181 (0.7%)	3,482 (4.0%)
Congestive heart failure	4,635 (1.3%)	392 (0.4%)	2,144 (1.6%)	135 (0.5%)	1,964 (2.3%)
Transient ischemic attack	1,435 (0.4%)	133 (0.1%)	663 (0.5%)	43 (0.2%)	596 (0.7%)
Venous thromboembolism	63 (0.0%)	5 (0.0%)	34 (0.0%)	4 (0.0%)	20 (0.0%)
End-stage renal disease	109 (0.0%)	5 (0.0%)	77 (0.1%)	0 (0.0%)	27 (0.0%)
Liver cirrhosis	754 (0.2%)	102 (0.1%)	399 (0.3%)	41 (0.2%)	212 (0.2%)
Chronic obstructive pulmonary disease	18,744 (5.3%)	4,042 (3.7%)	8,337 (6.4%)	980 (3.7%)	5,415 (6.3%)
Cancer	4,757 (1.3%)	965 (0.9%)	2,243 (1.7%)	233 (0.9%)	1,316 (1.6%)
Cardiac surgery	157 (0.0%)	9 (0.0%)	98 (0.1%)	3 (0.0%)	47 (0.1%)
Smoking					
Non-smoker	241,159 (68.1%)	79,800 (72.2%)	87,556 (67.1%)	16,871 (63.8%)	56,932 (65.8%)
Ex-smoker	18,470 (5.2%)	4,354 (3.9%)	7,058 (5.4%)	1,457 (5.5%)	5,601 (6.5%)
Current-smoker	94,454 (26.7%)	26,377 (23.9%)	35,969 (27.5%)	8,120 (30.7%)	23,988 (27.7%)
Drinking					
Non-drinker	185,788 (52.5%)	57,771 (52.3%)	70,104 (53.7%)	13,009 (49.2%)	44,904 (51.9%)
2–3 times per month	65,693 (18.6%)	25,528 (23.1%)	21,000 (16.1%)	5,545 (21.0%)	13,620 (15.7%)
≥ 1 time per week	102,602 (29.0%)	27,232 (24.6%)	39,479 (30.2%)	7,894 (29.8%)	27,997 (32.4%)
Exercise					
None	193,463 (54.6%)	63,808 (57.7%)	71,533 (54.8%)	13,494 (51.0%)	44,628 (51.6%)
1–4 times per week	131,245 (37.1%)	39,896 (36.1%)	46,766 (35.8%)	10,965 (41.5%)	33,618 (38.9%)
≥ 5 times per week	29,375 (8.3%)	6,827 (6.2%)	12,284 (9.4%)	1,989 (7.5%)	8,275 (9.6%)
Low income (Lowest 20 percentile)	54,983 (15.5%)	17,313 (15.7%)	20,951 (16.0%)	3,867 (14.6%)	12,852 (14.9%)
Rural area	190,202 (53.7%)	57,071 (51.6%)	71,154 (54.5%)	14,265 (53.9%)	47,712 (55.1%)
Obesity	112,969 (31.9%)				
BMI 25–29.9 kg/m^2^	101,657 (28.7%)			24,861 (94.0%)	76,796 (88.8%)
BMI ≥ 30 kg/m^2^	11,312 (3.2%)			1,587 (6.0%)	9,725 (11.2%)
Metabolically unhealthy	217,104 (61.3%)				
Number of MetS criteria					
1			83,399 (63.9%)		44,091 (51.0%)
2			38,102 (29.2%)		31,576 (36.5%)
3			9,082 (7.0%)		10,854 (12.5%)
Mean follow-up duration, years	7.43 ± 1.52	7.45 ± 1.48	7.44 ± 1.54	7.45 ± 1.48	7.38 ± 1.55

MHNW = metabolically healthy normal weight; MUNW = metabolically unhealthy normal weight; MHO = metabolically healthy obese; MUO = metabolically unhealthy obese; BMI = body mass index; MetS = metabolic syndrome

* SBP ≥ 130 and/or DBP ≥ 85 mmHg;

^†^ Fasting glucose ≥ 100 mg/dL;

^‡^ Total cholesterol ≥ 240 mg/dL

### Incidence and risk of stroke according to obesity and metabolic healthiness

**Fig 1:**
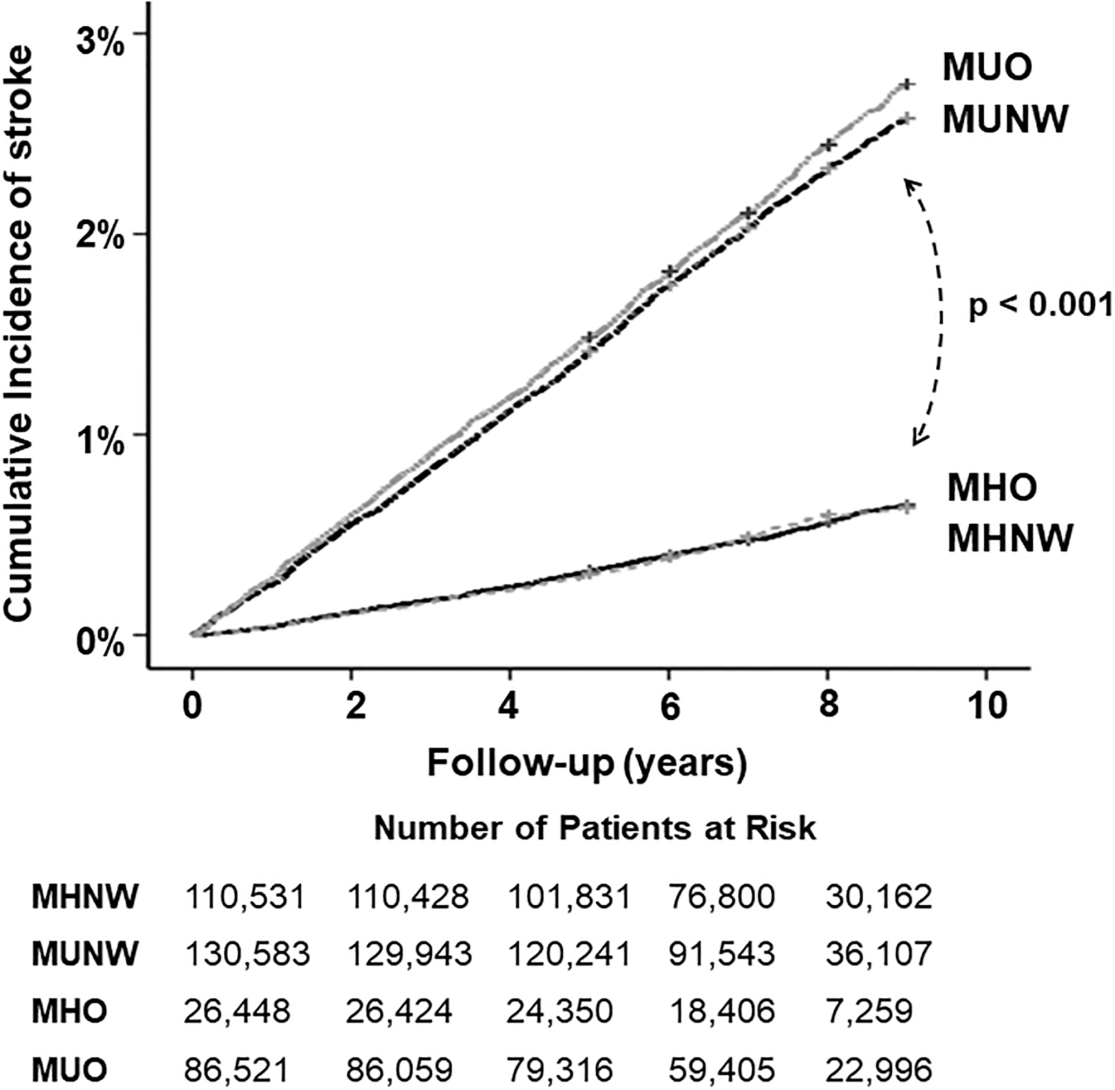
Kaplan-Meier curves showing the incidence of ischemic stroke according to groups categorized by obesity and metabolic health. MHNW = metabolically healthy normal weight; MUNW = metabolically unhealthy normal weight; MHO = metabolically healthy obese; MUO = metabolically unhealthy obese. P-value for comparison by log-rank test.

During mean follow-up of 7.43 years, (SD, 1.52 years), total 4,884 (1.4%) cases of newly diagnosed ischemic stroke were detected (incidence rate, 1.86 per 1,000 person-years). The cumulative incidence of ischemic stroke for each group are shown in Kaplan-Meier curves (Fig 1). Stroke incidence was significantly increased in the metabolically unhealthy groups (MUNW, MUO) compared to the metabolically healthy groups (MHNW, MHO) (p < 0.001 by log rank test), while there was no significant difference between the MHNW and MHO groups (p = 0.409) or between the MUNW and MUO groups (p = 0.064). Stroke incidence rates were nearly same in metabolically healthy MHNW and MHO groups (0.56 and 0.61 per 1,000 person-years), while they were more than four-fold higher in metabolically unhealthy MUNW and MUO groups (2.61 and 2.76 per 1,000 person-years) (Table 2). Risk for ischemic stroke was not increased in MHO compared to MHNW individuals, both before and after multivariate adjustment (adjusted HR 0.99, 95%CI 0.81–1.20), and in both younger and older ages. Meanwhile, stroke risk was significantly increased in both metabolically unhealthy groups (MUNW and MUO) compared to MHNW individuals.

**Table 2:** Incidence and risk of ischemic stroke according to groups divided by obesity and metabolic health.

	MHNW (n = 110,531)	MUNW (n = 130,583)	MHO (n = 26,448)	MUO (n = 86,521)
Stroke cases, n (%)	465 (0.4%)	2,535 (1.9%)	121 (0.5%)	1,763 (2.0%)
Stroke incidence (per 1,000 person-years)	0.56	2.61	0.61	2.76
Hazard ratio (HR)				
Crude^†^ HR (95% CI)	1 (ref)	4.62 (4.19–5.10)	1.09 (0.89–1.33)	4.90 (4.42–5.42)
Adjusted^‡^ HR (95% CI)	1 (ref)	1.72 (1.55–1.90)	0.99 (0.81–1.20)	2.06 (1.85–2.28)
Age < 50	1 (ref)	1.57 (1.25–1.95)	1.01 (0.70–1.46)	2.38 (1.90–2.97)
Age ≥ 50	1 (ref)	1.64 (1.46–1.84)	0.94 (0.74–1.20)	1.87 (1.66–2.10)

^†^ Unadjusted crude hazard ratio (HR) and 95% confidence interval (CI);

^‡^ Adjusted for age, sex, income, area, smoking, drinking, exercise, history of ischemic heart disease, peripheral artery disease, congestive heart failure, transient ischemic attack, venous thromboembolism, chronic obstructive pulmonary disease, end-stage renal disease, liver cirrhosis, cancer, and cardiac surgery

### Risk of stroke according to BMI and number of metabolic risk factors

**Fig 2:**
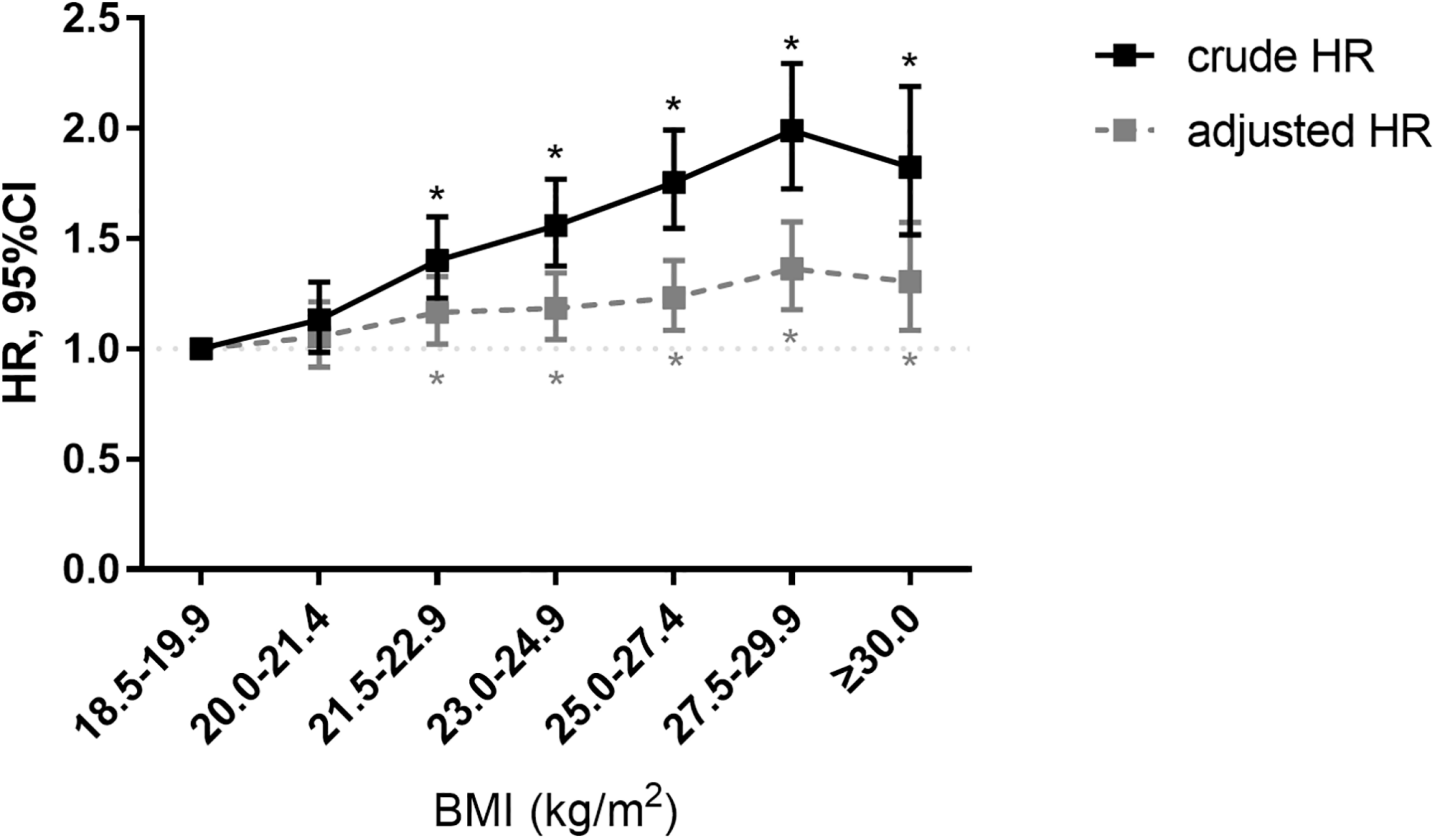
Risk for ischemic stroke according to BMI. BMI = body mass index. Each point representing hazard ratios (HRs) with error bars for 95% confidence intervals (95% CI). Asterisks indicated significant difference with reference group.

**Fig 3:**
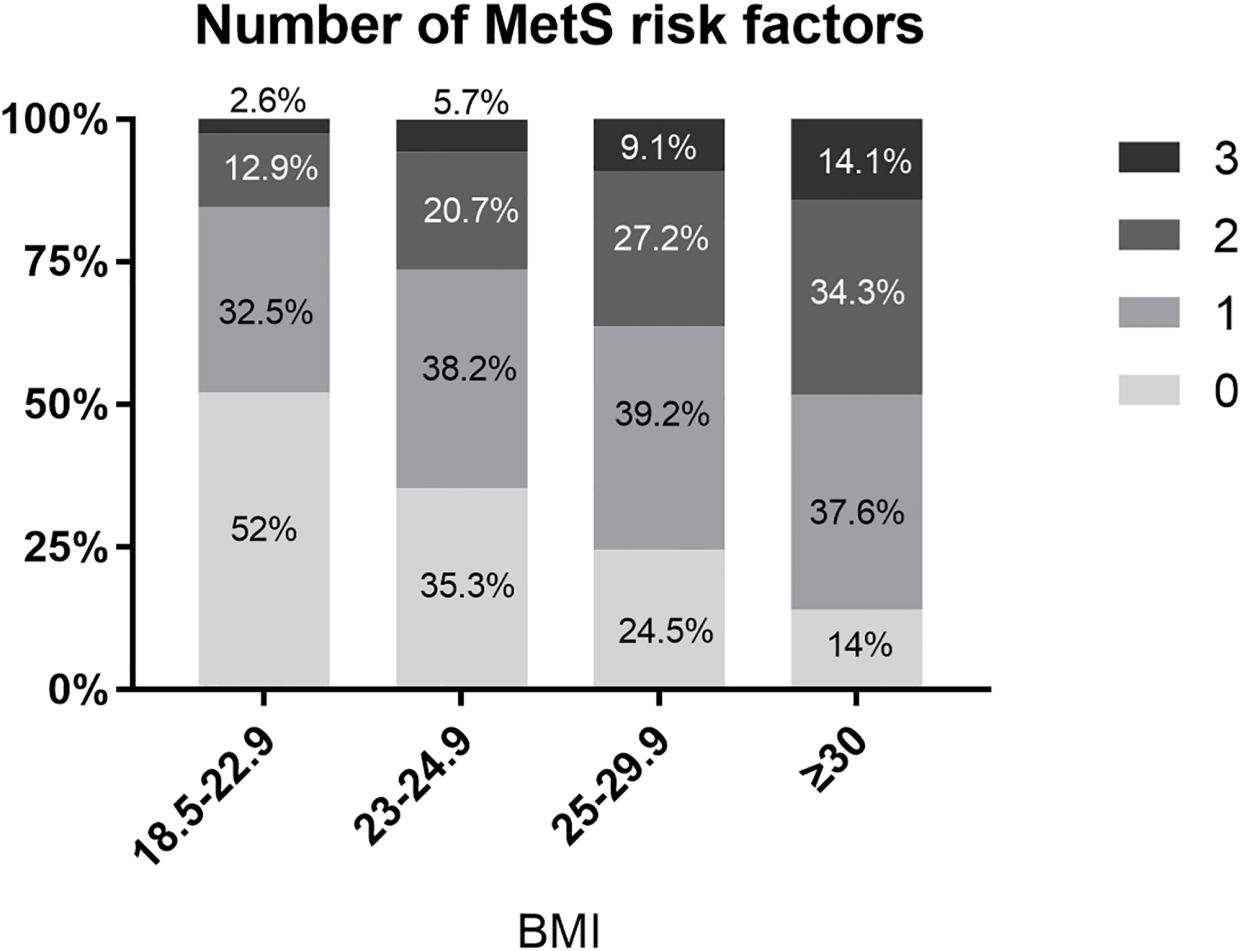
Number of metabolic syndrome risk factors according to BMI intervals. MetS = Metabolic syndrome; BMI = body mass index.

Table 3 presents the association of obesity, metabolic health components, and metabolic health status with ischemic stroke. Compared to normal weight individuals, obese individuals had 16% increased risk of ischemic stroke (HR 1.16, 95%CI 1.09–1.23), which was significant after adjustment for other metabolic syndrome criteria, i.e. elevated blood pressure, glucose, or cholesterol levels (Table 3, Model 2). This was true for both stage I and stage II obesity. The association with stroke became stronger with increasing BMI, though the association was much attenuated after adjustment for metabolic health components (Fig 2). The number of metabolic syndrome risk factors increased in proportion to higher BMI interval (Fig 3).

**Table 3:** The association of obesity, metabolic health components, and metabolic health status with ischemic stroke.

	N	Stroke cases	Model 1^†^ HR (95% CI)	Model 2^‡^ HR (95% CI)	Model 3^§^ HR (95% CI)
Obesity					
Normal weight	241,114	3,000 (1.2%)	1 (reference)	1 (reference)	
Obese	112,969	1,884 (1.7%)	1.25 (1.18–1.32)	1.16 (1.09–1.23)	
Stage I obesity (BMI 25–29.9 kg/m^2^)	101,657	1,700 (1.7%)	1.24 (1.17–1.31)	1.15 (1.09–1.23)	
Stage II obesity (BMI ≥ 30 kg/m^2^)	11,312	184 (1.6%)	1.37 (1.18–1.59)	1.21 (1.04–1.41)	
Metabolic health components (MetS criteria)					
Elevated blood pressure or treatment for hypertension	161,450	3,784 (2.3%)	1.78 (1.66–1.91)		1.74 (1.62–1.86)
Elevated glucose or treatment for diabetes mellitus	101,374	2,314 (2.3%)	1.25 (1.18–1.33)		1.24 (1.17–1.32)
Elevated total cholesterol or treatment for dyslipidemia	63,830	1,567 (2.5%)	1.23 (1.15–1.31)		1.22 (1.14–1.30)
Metabolic health status					
Healthy (0 MetS criteria)	136,979	586 (0.4%)	1 (reference)		1 (reference)
Unhealthy (≥1 MetS criteria)	217,104	4,884 (1.4%)	1.86 (1.70–2.03)		1.80 (1.65–1.97)

HR = Hazard ratio; CI = Confidence interval; BMI = body mass index; MetS = metabolic syndrome

^†^ Adjusted for age, sex, income, area, smoking, drinking, exercise, history of ischemic heart disease, peripheral artery disease, congestive heart failure, transient ischemic attack, venous thromboembolism, chronic obstructive pulmonary disease, end-stage renal disease, liver cirrhosis, cancer, and cardiac surgery;

^‡^ Further adjustment for metabolic health components;

^§^ Further adjustment for obesity

The association of metabolic unhealthiness with stroke appeared more clearly in analysis stratified by BMI groups (Table 4). In both normal weight and obese individuals, the metabolically unhealthy were at increased risk of ischemic stroke compared to the metabolically healthy (for normal weight: HR 1.68, 95%CI 1.52–1.87; for obese: HR 2.16, 95%CI 1.79–2.60). The association seemed to become stronger with increasing degree of obesity.

**Table 4:** The association of metabolic health status with ischemic stroke stratified by body mass index groups.

	N	Stroke cases	HR (95% CI)
Normal weight (BMI 18.5–24.9 kg/m2)			
Metabolically healthy	110,531	465 (0.4%)	1 (reference)
Metabolically unhealthy	130,583	2,535 (1.9%)	1.68 (1.52–1.87)
Obese (BMI ≥ 25 kg/m^2^)			
Metabolically healthy	26,448	121 (0.5%)	1 (reference)
Metabolically unhealthy	86,521	1,763 (2.0%)	2.16 (1.79–2.60)
Stage I obesity (BMI 25–29.9 kg/m^2^)			
Metabolically healthy	24,861	115 (0.5%)	1 (reference)
Metabolically unhealthy	76,796	1,585 (2.1%)	2.13 (1.76–2.59)
Stage II obesity (BMI ≥ 30 kg/m^2^)			
Metabolically healthy	1,587	6 (0.4%)	1 (reference)
Metabolically unhealthy	9,725	178 (1.8%)	2.32 (1.02–5.29)

HR = Hazard ratio; CI = Confidence interval; BMI = body mass index

Adjusted for age, sex, income, area, smoking, drinking, exercise, history of ischemic heart disease, peripheral artery disease, congestive heart failure, transient ischemic attack, venous thromboembolism, chronic obstructive pulmonary disease, end-stage renal disease, liver cirrhosis, cancer, and cardiac surgery

All components of metabolic syndrome criteria were associated with incident stroke as well, which was significant after adjustment for obesity (Table 3, Model 3). Elevated blood pressure showed the strongest association with stroke development (HR 1.74, 95%CI 1.62–1.86). Compared with metabolically healthy individuals, metabolically unhealthy individuals had an 80% increased risk for ischemic stroke with adjustment for obesity (HR 1.80, 95%CI 1.65–1.97). Overall, the presence of each metabolic syndrome criteria or metabolic unhealthiness showed stronger association with ischemic stroke than obesity status.

Meanwhile, the association of obesity with ischemic stroke differed according to metabolic health status (Table 5). In metabolically healthy individuals, obesity did not increase the risk for ischemic stroke (HR 1.00, 95%CI 0.81–1.22), regardless of the severity of obesity. Meanwhile, in metabolically unhealthy individuals, being obese was significantly associated with increased risk of stroke (HR 1.19, 95%CI 1.12–1.27), for both stage I and II obesity.

**Table 5:** The association of obesity with ischemic stroke stratified by metabolic health status.

	N	Stroke cases	HR (95% CI)
Metabolically healthy			
Normal weight (BMI 18.5–24.9 kg/m2)	110,531	465 (0.4%)	1 (reference)
Obese (BMI ≥ 25 kg/m^2^)	26,448	121 (0.5%)	1.00 (0.81–1.22)
Stage I obesity (BMI 25–29.9 kg/m^2^)	24,861	115 (0.5%)	0.99 (0.81–1.22)
Stage II obesity (BMI ≥ 30 kg/m^2^)	1,587	6 (0.4%)	1.06 (0.47–2.38)
Metabolically unhealthy			
Normal weight (BMI 18.5–24.9 kg/m2)	130,583	2,535 (1.9%)	1 (reference)
Obese (BMI ≥ 25 kg/m2)	86,521	1,763 (2.0%)	1.19 (1.12–1.27)
Stage I obesity (BMI 25–29.9 kg/m^2^)	76,796	1,585 (2.1%)	1.18 (1.11–1.26)
Stage II obesity (BMI ≥ 30 kg/m^2^)	9,725	178 (1.8%)	1.25 (1.07–1.46)

HR = Hazard ratio; CI = Confidence interval; BMI = body mass index

Adjusted for age, sex, income, area, smoking, drinking, exercise, history of ischemic heart disease, peripheral artery disease, congestive heart failure, transient ischemic attack, venous thromboembolism, chronic obstructive pulmonary disease, end-stage renal disease, liver cirrhosis, cancer, and cardiac surgery

## Discussion

### Stroke risk associated with obesity differs according to metabolic health status

The main finding of this study was that MHO individuals did not show increased risk for ischemic stroke compared to healthy normal weight individuals, while metabolically unhealthy individuals, both normal weight and obese, showed increased risk for stroke. While obesity appears to be significantly associated with ischemic stroke, this effect may differ according to metabolic health status: being obese increased risk for stroke in metabolically unhealthy individuals, but not in metabolically healthy individuals. Meanwhile, all metabolic health components showed stronger association with stroke than obesity, and being metabolically unhealthy increased risk for stroke in all BMI categories.

Obesity is associated with an increased risk for stroke. We used BMI as a measure of obesity, which is easily obtained and routinely measured in large population studies, and while it is moderately strongly correlated (30–50%) with fat-free mass, it is much more strongly correlated (60–90%) with fat mass and also (80–85%) with waist circumference[1]. Obesity was associated with 40% increased stroke mortality with each 5-kg/m^2^ increase in the BMI range of 25–50 kg/m^2^[1]; in a meta-analysis, the relative risk for ischemic stroke was 1.22 (95% CI, 1.05–1.41) for overweight and 1.64 (95% CI, 1.36–1.99) for obesity[2]. However, there is conflicting data on whether obese individuals without metabolic syndrome risk factors also have increased risk for stroke. One study of 5,171 subjects with 9.1 years of follow-up found that MHO did not show greater risk for ischemic stroke compared to metabolically healthy non-obese subjects (HR 1.07, 95%CI 0.93–1.24)[14], even though MHO was defined as having up to 1 risk factors. Another study that followed 19,675 participants for 18.7 years, found that MHO (having 0 risk factors) did not have increased risk for not only stroke, but coronary heart disease and overall mortality as well, and only showed moderate increase in risk for diabetes[15]. Other studies that included stroke as part of cardiovascular outcome found that MHO did not show difference in risk compared to the metabolically healthy non-obese [16, 17]. On the other hand, the Whitehall II study followed up 7,122 subjects for 17.4 years, and found that MHO subjects were at increased risk for cardiovascular disease including stroke (HR 1.95, 95%CI 1.37–2.77), and that stroke analyzed separately showed similar results[11]. However, this study defined MHO as having 0–1 risk factor, and stroke included cerebral hemorrhage as well as ischemic stroke. Some other studies including stroke in cardiovascular outcome also found that risk was increased in MHO compared to MHNW[12, 13].

### MHO and cardiovascular risk under debate

The existence of a MHO phenotype with lower cardiovascular risk remains under debate. The inconsistent evidence may be related to several factors. First, the definition of metabolic health is very important and different definitions may lead to different results. Metabolic syndrome is defined as having 3 or more of 5 risk factors, and so many have considered metabolically healthy to be its opposite, i.e. having 1 or less of 4 risk factors (excluding waist circumference). However, it has been argued that a person with even 1 risk factor, such as impaired glucose tolerance or treatment for hypertension, cannot be considered truly metabolically healthy[9]. Many previous studies defined MHO as obese persons with up to 1 metabolic risk factor, and including these people may have confounded results by increasing cardiovascular risk for the thus defined MHO group. According to a recently proposed harmonized definition of MHO[9], a person should have none of the metabolic syndrome risk factors to be considered metabolic healthy. We adopted this stricter definition restricting MHO to those who are obese but fully healthy from a metabolic point of view. In a previous study, when defining MHO as being obese and having 0 risk factors, the prevalence of MHO among the obese was 16.6%, while it increased nearly double-fold to 31.7% when those having up to 1 risk factor were included[31]. In another large study defining MHO as meeting 0 metabolic syndrome criteria, the prevalence of MHO was 7–28% in women, and 2–19% in men[32]. In our cohort, a third (31.9%) were obese, of whom a fourth (23.4%) were metabolically healthy; thus, 7.5% of the population were MHO.

Second, the prognosis of MHO may be outcome specific; for example, cardiovascular disease and type 2 diabetes showed different associations[11, 16]. Coronary heart disease and ischemic stroke are often studied as a composite cardiovascular outcome, but may have different associations with obesity and metabolic health components, leading to inexact results. In some previous studies where MHO showed increased cardiovascular risk, the relative risk for stroke was less than that for coronary heart disease[15]. We recently reported that MHO individuals are at increased risk of atrial fibrillation (AF), and obesity is independently associated with AF development[18]. Despite the increased risk of AF, in this study, we found that MHO individuals did not show an increased risk of stroke compared to healthy non-obese individuals. Stroke risk may also differ depending on type of stroke, i.e. ischemic or hemorrhagic[33–35]; however, most studies on MHO did not differentiate types of stroke in the outcome[11–13, 15, 17]. Focusing on a single outcome would be ideal, if the study population was large enough. In this study, we confined the primary outcome to ischemic stroke in a population sufficiently large to obtain meaningful results.

### Study strengths and limitations

Several notable strengths of our study include its large sample size of over 354,000 adults, representability of the general Korean population, and assessment of outcomes over a more than 7-year follow-up period. All records of medical treatment were available for each individual in the cohort, leading to greater accuracy in detection of the primary outcome. In addition, a strict definition of MHO was used, making it possible to follow the prognosis of the obese who are fully metabolically healthy. Also, we focused on a single outcome instead of a composite cardiovascular outcome.

Our study also has some limitations. First, comorbidities were identified by diagnosis codes included in claims data, with a minimum requirement of number of outpatient or inpatient treatments. This relies on the assumption that the physician entered the correct diagnosis for each patient. Second, only total cholesterol levels were available and we used this as substitute for triglyceride and HDL levels in metabolic health criteria. Third, as the study cohort was followed up for a mean period of 7–8 years, it would be unable to detect an increase in the primary outcome if it happened after a lag of 10–15 years. However, in the above-mentioned Whitehall study, Kaplan-Meier survival curves for outcome diverged from the start of the study, not after a lag time[11]. Also in our previous study, 25% of the MHO group and 15% of the MHNW group were shown to become metabolically unhealthy after 7.5 years of follow-up, and these subgroups showed increased risk for AF[18]. Thus, a longer follow-up period may lead to a higher portion of initially metabolically healthy groups becoming metabolically unhealthy and thereby showing an increase in outcome. Fourth, as the cohort was homogeneously comprised of Koreans, there may be limitations in generalizing our findings to other ethnicities.

### Conclusion

In conclusion, obesity increased risk for ischemic stroke in the metabolic unhealthy, while not in the metabolically healthy. Obesity appears to be harmful especially in persons with metabolic risk factors, and maintaining normal weight may be more important for this population. On the other hand, MHO individuals were not at increased risk for ischemic stroke, and metabolically healthy obesity may be benign, at least in the case of ischemic stroke. Better understanding of the health risks associated with obesity can help physicians discern patients who should engage in weight reduction more actively. Also, metabolic unhealthiness showed greater association than obesity with stroke, stressing the importance of controlling metabolic risk factors.

## Supporting information

S1 Table International Classification of Disease (ICD) 10 codes and procedure codes used for variables. (DOCX)
