# Supplementary material for: Risk of ischemic stroke in metabolically healthy obesity: A nationwide population-based study
Source: PLoS One. 2018 Mar 30;13(3):e0195210. doi: 10.1371/journal.pone.0195210 (PMC5877885; doi:10.1371/journal.pone.0195210)
Supplement: S1 Table — (DOCX) [file pone.0195210.s001.docx]

**Supplementary Table 1. International Classification of Disease (ICD) 10 codes and procedure codes used for variables**

| Variables | ICD-10 codes | Procedure codes | Requisites |
| --- | --- | --- | --- |
| Hypertension | I10-15 |  | * |
| Diabetes mellitus | E11-14 |  | † and prescription of hypoglycemic drugs |
| Dyslipidemia | E78 |  | † |
| Ischemic heart disease | I20-25 |  | * |
| Peripheral artery disease | I70, I73 |  | * |
| Congestive heart failure | I50 |  | †, in the previous 3 years |
| Transient ischemic attack | G458, G459 |  | † |
| Venous thromboembolism | I26, I802 |  | ≥ 1 diagnosis during hospitalization, in the previous 1 year |
| End-stage renal disease | N18, N19, Z49, Z905, Z94, Z992 | R3280 (kidney transplantation),  O7011-7020 (hemodialysis),  O7017, O7075 (peritoneal dialysis) | ≥ 1 diagnosis during hospitalization or at outpatient clinic and ≥ 1 procedure code, in the previous 3 years |
| Liver cirrhosis | K703, K743-745, K76 |  | ≥ 1 diagnosis during hospitalization, in the previous 1 year |
| Chronic obstructive pulmonary disease | J43-44 |  | † |
| Cancer | C00-97 |  | * |
| Cardiac surgery |  | O1660, O1671-1672, O1680, O1690, O1701-1705, O1710-1711, O1721-1723, O1730, O1740, O1750, O1760, O1770, O178-1783, O1791-O1798, O1800, O1810, O1821-1826, O1830, O1840-1844, O1850, O1861, O1873-1875, O1878-1879, O1890, O1895, O1901-1902, O1910, O1921-1922, O1931-1932, O1935, O1940, O1950, O1960, O1970, O1981-1982, O2001-2002, O2004-2007 | † |

* ≥ 1 diagnosis during hospitalization, or ≥ 2 diagnoses at outpatient clinic, in the previous 1 year

† ≥ 1 diagnosis during hospitalization or at outpatient clinic, in the previous 1 year
